# Surgical Interventions for Advanced Parameningeal Rhabdomyosarcoma of Children and Adolescents

**DOI:** 10.7759/cureus.2045

**Published:** 2018-01-09

**Authors:** Paul J Choi, Joe Iwanaga, R. Shane Tubbs, Emre Yilmaz

**Affiliations:** 1 Clinical Anatomy, Seattle Science Foundation; 2 Seattle Science Foundation; 3 Neurosurgery, Seattle Science Foundation; 4 Swedish Medical Center, Swedish Neuroscience Institute

**Keywords:** rhabdomyosarcoma, soft tissue sarcoma, childhood, adolescence, head and neck, parameningeal, skull base surgery, endoscopic, reconstruction, microsurgery

## Abstract

Owing to its rarity, rhabdomyosarcoma of the head and neck (HNRMS) has seldom been discussed in the literature. As most of the data is based only on the retrospective experiences of tertiary healthcare centers, there are difficulties in formulating a standard treatment protocol. Moreover, the disease is poorly understood at its pathological, genetic, and molecular levels. For instance, 20% of all histological assessment is inaccurate; even an experienced pathologist can confuse rhabdomyosarcoma (RMS) with neuroblastoma, Ewing’s sarcoma, and lymphoma. RMS can occur sporadically or in association with genetic syndromes associated with predisposition to other cancers such as Li-Fraumeni syndrome and neurofibromatosis type 1 (von Recklinghausen disease). Such associations have a potential role in future gene therapies but are yet to be fully confirmed. Currently, chemotherapies are ineffective in advanced or metastatic disease and there is lack of targeted chemotherapy or biological therapy against RMS. Also, reported uses of chemotherapy for RMS have not produced reasonable responses in all cases. Despite numerous molecular and biological studies during the past three decades, the chemotherapeutic regimen remains unchanged. This vincristine, actinomycin, cyclophosphamide (VAC) regime, described in Kilman, et al. (1973) and Koop, et al. (1963), has achieved limited success in controlling the progression of RMS. Thus, the pathogenesis of RMS remains poorly understood despite extensive modern trials and more than 30 years of studies exploring the chemotherapeutic options. This suggests a need to explore surgical options for managing the disease. Surgery is the single most critical therapy for pediatric HNRMS. However, very few studies have explored the surgical management of pediatric HNRMS and there is no standard surgical protocol. The aim of this review is to explore and address such issues in the hope of maximizing the number of options available for young patients with HNRMS.

## Introduction and background

Rhabdomyosarcoma (RMS) is a rare high-grade soft tissue sarcoma of mesenchymal origin, with evidence of striated muscle cell differentiation [1-7]. It represents 5%-7% of all pediatric malignancies and is the most prevalent sarcoma of childhood and adolescence, i.e., 50% of all reported sarcoma cases [1, 2, 4, 8-11]. It is also the third most common pediatric extracranial solid tumor after neuroblastoma and Wilms’s tumor [2, 12-13].

RMS lacks a capsule, a membranous envelope [8, 14-15]. This allows it to expand rapidly and engulf regional structures, so there are early and considerable local invasions and significant metastatic potential [2-4, 7, 9, 14, 16-18]. RMS also has a local relapse rate of 20%-40%, which contributes to the dismal five-year survival rate of 24%-30% [1, 2, 8, 10, 12-16, 19]. Relapse precedes rapid development of a metastatic disease [14]. Thus, diagnosis of RMS needs to be timely, and a targeted therapeutic protocol should be initiated promptly for an optimal outcome [18, 20-21]. However, this is seldom achieved; 50% of all RMS cases are high-grade, locally advanced, or even metastatic at the time of diagnosis [22].

Most RMS in the pediatric population (30%-40%) occurs in the head and neck region [2-3, 8-11, 16-17, 23-24]. Between 1975 and 2005, there were significant and unexplained increases in the annual incidences of rhabdomyosarcoma of the head and neck (HNRMS) by 1.16%, and of alveolar RMS, mostly HNRMS, by 4.2% [16]. Pediatric HNRMS has an overall survival rate of 28.7% [16]. 44% percent of all HNRMS occurs in the parameningeal region: paranasal sinuses, nasal cavity, nasopharynx, middle ear, and the skull base [8, 25-26].

Parameningeal location has the least favorable prognosis (Table 1) [8]. This is due to not only the complexity of its anatomy and its proximity to the cranial cavity, and potential dissemination via the cerebrospinal fluid (CSF), but also to the paucity of distinctive symptoms (it can even be asymptomatic in early stages) (Table 2) [9, 20, 25]. Symptoms often mimic chronic upper respiratory tract infection (URTI), otitis media (OM), and soft tissue injury (nasal discharge and congestion, otorrhea, and mild swelling) [2]. Such characteristics of this location increase the risk of misdiagnosis and of diagnosis delayed by up to a month [8-9, 18, 26-27]. Late diagnosis of HNRMS is directly associated with poor prognosis [20].

**Table 1:** Pediatric RMS locations RMS; Rhabdomyosarcoma

Pediatric RMS Locations
Favorable: Orbit(s) Non-Parameningeal Head and Neck Region Genitourinary (Non-Bladder, Non-Prostate) Biliary Tract​​​​​​​
Unfavorable: Bladder/Prostate Extremity Cranial Parameningeal (most common) Other (Trunk, Retroperitoneum, etc.)

**Table 2:** Characteristics of pediatric HNRMS HNRMS; rhabdomyosarcoma of the head and neck, URTI; upper respiratory tract infection, OM; otitis media

Pediatric HNRMS Characteristics	Description
Histology	Lacks a Capsule [8, 14, 15]
Most Common Location	Parameningeal [8, 25, 26]
Presentation	Very few symptoms mimicking URTI, OM, and Soft-Tissue Injury [2]
Rate of Advanced Cancer at the Time of Diagnosis	50% [22]
Misdiagnosis/Delayed Diagnosis Rate	High [8, 9, 18, 26, 27]
Rate of Growth	Rapidly Progressive [2, 4, 7, 9, 16]
Recurrence Rate	20% – 40% [8, 10, 12, 13, 16]
Five-year Survival Rate Post-recurrence	24% – 30% [1, 2, 8, 14-16, 19]
Metastatic Potential	Rapidly Progress to Metastatic Disease Post-recurrence [14]

Surgery is the mainstay of managing parameningeal HNRMS [28]. However, surgical resection is challenging owing to the high likelihood of a disease-positive margin post-resection and risk of injury to the brain parenchyma because of its anatomical proximity and complexity [8, 22, 25-27]. Moreover, the role of surgery has been deemed limited in the pediatric population because it is more difficult to achieve suitable surgical access, and functional and cosmetic morbidities often follow [25-26]. However, innovations in craniofacial operation and reconstruction techniques, as an element of the multimodal, multidisciplinary protocol developed by the Intergroup RMS Study Group (IRSG) and Children’s Oncology Group (COG), have yielded satisfactory results in recent years [25]. This protocol focuses mainly on non-surgical interventions.

## Review

Limitations of current chemotherapeutic and potential novel biological therapy regimens

Intergroup RMS Study Group (IRSG) and Children’s Oncology Group (COG) previously conducted clinical trials to assess the efficacy of adding agents such as doxorubicin, etoposide, ifosfamide, and irinotecan to the vincristine, actinomycin, cyclophosphamide (VAC) regime, and even attempted to increase the dose of cyclophosphamide in the hope of achieving better prognoses [2, 29]. However, none of these endeavors led to a significant improvement [2, 29].

**Figure 1:**
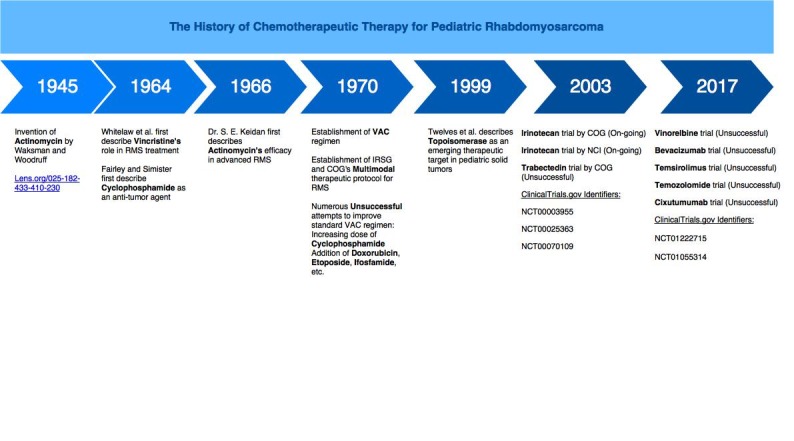
The chronology of chemotherapeutic and biological regimens for pediatric RMS RMS; rhabdomyosarcoma, VAC; vincristine/actinomycin/cyclophosphamide, IRSG; Intergroup RMS Study Group, COG; Children’s Oncology Group, NCI; National Cancer Institute

In recent years, extensive studies exploring potential novel molecular targets have been conducted on animal models, including genetically-modified and xenograft models [5, 8, 30-31]. These were followed by comprehensive clinical trials to assess the efficacies of insulin-like growth factor (IGF1) receptor inhibitors such as cixutumumab (www.clinicaltrials.govNCT00831844); the vascular endothelial growth factor (VEGF) inhibitor sorafenib (www.clinicaltrials.govNCT01502410); granulocyte-macrophage colony-stimulating factor (GM-CSF) inhibitors such as sargramostim (www.clinicaltrials.govNCT00003955, NCT00002995, NCT00003597,NCT00025363, NCT00003958); and epidermal growth factor receptor (EGFR) inhibitors such as erlotinib [8]. Unfortunately, none of these trials demonstrated the novel agents’ clinical efficacies and some were prematurely terminated owing to patients’ inability to tolerate the protocol (Figure 1) [1, 5, 12, 30, 32]. The value of chemotherapy and biological therapy in managing rhabdomyosarcoma (RMS) remains unclear [31-32].

Current multi-modal, multi-disciplinary protocol for managing RMS

The rarity of RMS and the poor understanding of its pathogenesis have encouraged international research groups, i.e., IRSG and COG, to develop a stratification system to assess prognosis and to formulate standardized therapeutic protocols to introduce more targeted management [8].

Since the 1970s, using extensive analyses of clinical data, IRSG has developed a multidisciplinary stratification system that categorizes RMS into Group I (a localized disease that is completely resected) to IV (metastatic disease) [5, 8, 30, 31]. This system has allowed highly individualized multimodal therapeutic protocols, i.e., combinations of surgery, radiotherapy, and chemotherapy [5, 8, 16, 20, 26], to be integrated into RMS management and has contributed to the increase in five-year survival rate from 25% in 1970 to 87% [2, 3, 5, 8, 14, 16, 17, 30]. However, the 87% relates to a patient group with confined, favorable location [9, 12]. It is necessary to explore the efficacy of such protocols in groups with unfavorable factors (five-year survival rate of 31%) i.e., parameningeal location, alveolar histology, >10 years of age, incomplete surgical resection prior to adjuvant systemic treatment, and regional lymph node infiltration [8, 18, 22, 30]. The IRSG/COG clinical grouping system is depicted in Table 3. The clinical groups are assigned at diagnosis, i.e., after initial surgical management aimed at achieving wide local excision with a disease-negative margin and providing the most accurate prediction of treatment prognosis [31]; “operability” is the single most significant influence on patient survival (Table 3) [15].

**Table 3:** COG/IRSG clinical group stratification system for RMS RMS; rhabdomyosarcoma, COG; Children’s Oncology Group, IRSG; Intergroup RMS Study Group

Group	Definition	Survival rate
I	Completely resected localized tumor	87%
IIA	Grossly resected tumor WITH microscopic residual disease	≤ 31%
IIB	Completely resected involved regional nodes with NO microscopic residual disease	31% – 87%
IIC	Grossly resected involved regional nodes WITH microscopic residual disease	≤ 31%
III	Incompletely resected tumor WITH gross residual disease OR biopsy-only	≤ 31%
IV	Distant metastasis	0%

Challenges of approaching HNRMS surgically:

Surgical management of parameningeal HNRMS: conservative or revolutionary

From reviewing numerous articles, we concluded that there are two conflicting views amongst surgeons on the surgical management of parameningeal HNRMS: the conservative and the revolutionary. 

Conservative

The traditional surgical intervention for RMS has been extensive, wide local resection with concomitant removal of a 0.5 cm thick envelope of normal tissue to achieve a clear disease-negative margin and to preserve the functions of nearby vital structures [10, 15, 31]. The aim of RMS surgery has been to perform a “curative,” complete resection to achieve the 80% overall survival rate [2, 6, 10]. However, it is often not possible to achieve such a margin in a complex anatomy such as that of the parameningeal region.

Resection of tumors in the head and neck region is especially challenging because of important tissues nearby such as neurovascular bundles and the brain, the complexity of the anatomy, and spatial restraints [14, 15, 19, 20, 28, 33]. Therefore, it is difficult to perform an ideal wide en bloc resection with a disease-negative margin without injuring adjacent structures [14, 15, 19, 20, 28, 33] and risking postoperative morbidities such as problems with vocalization, food ingestion, and respiration, and also serious cosmetic defects [14, 15, 19, 20, 28, 33].

Hence, a number of authors encourage abandonment of the surgical approach, except for excisional biopsy [8, 31], if the lesion is situated deep in the head and neck or if adequate surgical access is not attainable [8, 14], so functional and cosmetic defects that could affect quality of life adversely are avoided [34]. They also discourage the use of surgical debulking in the head and neck area [2, 29]. In addition, the COG recommends systemic therapy only for high-grade, locally advanced, relapsed, or metastatic disease [2, 29]. Patients undergoing resection of parameningeal HNRMS tend to be high-grade with a disease-positive margin [22]. As previously discussed, the grade is an important determinant of prognosis in RMS cases [22].

Revolutionary

In contrast, some authors advocate the use of challenging yet highly technical surgical procedures to achieve a disease-negative margin for parameningeal tumors and to accomplish “operability” by adopting a “surgical approach that is based on the individual characteristics of each patient” [26]. They endorse combined craniofacial and endoscopic resection and reconstructive surgery by a multidisciplinary team of laryngologists, maxillofacial surgeons, neurosurgeons, plastic surgeons, and ophthalmologists [8, 27], and further stretch their support of surgery to “palliative” operations.

Current surgical techniques for pediatric parameningeal HNRMS

Current surgical interventions for pediatric parameningeal HNRMS include wide local excision and endoscopic technology to achieve a disease-negative margin, reconstructive surgery including that for skull base defects, and application of micro-surgery. The aim is relapse-free remission of the disease and also a symptomatic relief for those undergoing chemotherapy and the terminally ill, ultimately improving the quality of life of the young population. The combination of technological advances and deep understanding of micro-anatomy of the head and neck allows for a safe operation [33].

Traditional wide local resection to achieve a disease-negative margin

This technique, when used in the head and neck area, can entail a risk of incomplete resection [13]. Residual disease places RMS in a high-grade category, i.e., Group II to IV [34]. Half of all high-grade tumors are followed by local recurrence and subsequent rapid progression into metastasis [13]. Thus, the surgeon should aim for a good 0.5 cm margin in the first procedure if possible [13]. Otherwise, second-look explorations may be necessary, especially if a gross residual mass remains unresected [14, 15, 18, 20, 31].

Furthermore, a complete wide local resection of parameningeal HNRMS with a disease-negative margin is often possible. For instance, Demonte, et al. describe a case of a 9.5-year-old boy with relapsed maxillary sinus RMS who underwent wide local resection with osteotomies, i.e., maxillectomy, mandibulectomy, and removal of the middle cranial fossa, and had complete remission of the disease [35].

On the other hand, Lindford, et al. claim that a disease-positive margin does not diminish survival rates in pediatric HNRMS if peri-operative chemoradiotherapies are performed [15]. In addition, the recurrence risk plummets with the use of systemic adjuvant therapy, which is recommended for all high-grade RMS [14, 30]. The prognosis is even more favorable if a second-look excision is performed and is followed by adjuvant systemic therapy [26, 31]. This surgical technique is referred to as “reasonable” excision, which “seems to be the best method of initiating therapy” for RMS [10] and yields a survival rate comparable to that of radical resection [2, 10].

Minimally invasive endoscopic resection (MIER) as an emerging surgical technique for HNRMS

Since the 1960s, craniofacial resection has been the surgical method of choice since it allows wide excision with a satisfactory margin [36]. However, the method commonly led to major functional and cosmetic morbidities [36], the most common functional defect being cerebrospinal fluid (CSF) leakage in up to 20% of all cases [36]. In contrast, MIER minimizes handling of the skull base and prevents the morbidity and further complications that conventional resection delivers, i.e., meningitis, cerebral abscess, pneumocephalus, brain herniation and, consequently, even death [36]. This reduction in morbidity not only improves quality of life, it also shortens the period of hospital stay and reduces healthcare costs [36-37].

MIER provides such benefits by using a microscope, which provides magnified vision, better lighting, and superior corner visualization, allowing vital structures to be identified in detail and hence ensuring their preservation [27, 36]. MIER allows for ideal exposure of the median and paramedian structures of the skull base including the cavernous sinus, once called “No Man’s Land” [27, 37]. Tumors invading the cavernous sinus have traditionally been resected radically via maxillectomy, the transcranial approach, and the petrosal approach; however, the resection can now be done endoscopically [37].

He, et al. support the view that MIER is superior to conventional craniofacial resection in reducing recurrence rate and improving overall survival in carefully-selected parameningeal HNRMS cases [36, 38]. Their study also reported that only three out of 120 enrolled patients showed post-operative complications, which were conservatively treated [36]. It also describes a case of a locally advanced ethmoid sinus RMS invading the skull base, which was successfully cured by MIER [36]. In addition, Bostanci, et al. report another case of parameningeal RMS in a two-year-old, which was completely resected by MIER with a margin free of disease [18].

MIER also allows chemotherapy to be resumed immediately postoperatively [28]. When MIER was combined with perioperative chemoradiotherapies, the recurrence rate was lower and the outcome was better than those of conventional surgery [26]. Moreover, the technology has a major role in skull base reconstruction for repairing CSF leaks with fibrin glue, hydroxyapatite cement, or autografts [15, 35, 39, 40]. The summary of the comparison between the traditional wide local resection and MIER is summarized in Table 4.

**Table 4:** Comparison of traditional wide local resection and minimally invasive endoscopic resection in HNRMS HNRMS; rhabdomyosarcoma of the head and neck

	Wide Local Resection	Minimally Invasive Endoscopic Resection
Disease Negative Margin Achievability	Satisfactory [13]	Satisfactory [36]
Tissue Preservation	Inferior [27, 36]	Superior [27, 36]
Chemotherapy Resumption	Delayed [28]	Immediate [28]
Functional and Cosmetic Morbidities	More [36]	Fewer [36]
Overall Quality of Life	Inferior [36]	Superior [36]
Length of Hospital Stay	Longer [36, 37]	Shorter [36, 37]
Healthcare Cost	More [36, 37]	Less [36, 37]
Application in Skull Base Surgery	No	Yes [15, 35, 39, 40]

Reconstructive surgery as an adjunct to a radical resection to minimize structural defects

Regardless of the method, i.e., conventional wide local resection, MIER, or a combination of both, radical resection should be followed by reconstruction to restore significant functional and anatomical defects using tissue flaps: regional or free [8, 30].

Reconstruction of a defect is especially crucial in pediatric patients since they are more vulnerable to psychological trauma and are exposed to a lifetime risk of morbidities [1]. However, a reconstructive operation is especially difficult to perform for them since they are in a growth phase and therefore susceptible to retraction deformity and severe post-reconstruction donor site morbidity [41].

Flaps used in pediatric head and neck reconstructive surgery

It has been reported that pediatric patients are at greater risk of developing post-operative complications with free flap use [41]. Hence, regional flaps are recommended whenever feasible. However, the use of free flaps becomes inevitable when larger defects are repaired [15]. Weizman, et al. describe such flaps as effective and safe for HNRMS patients who have undergone a radical operation [15, 28, 39]. In addition, free flaps show superior healing, functional revitalization, and esthetic outcome (Table 5) [39]. For example, Ueda, et al. reported a case of a 14-year-old girl whose big skin defect post-HNRMS-resection was reconstructed with a free latissimus dorsi muscle flap, after which she was able to eat normally at week 12 and remained disease-free at year four with a reported good quality of life [42].

**Table 5:** Comparison of regional and free flaps for use in pediatric HNRMS HNRMS; rhabdomyosarcoma of the head and neck

	Regional Flaps	Free Flaps
Postoperative Complications	Fewer [41]	More [41]
Defect coverage	Covers Small Defects [15]	Covers Large Defects [15]
Healing Quality	Inferior [39]	Superior [39]
Cosmetic Outcome	Inferior [39]	Superior [39]
Functional Restoration	Inferior [39]	Superior [39]

Some examples of free flaps include: small bowel and forearm flaps for defects of moving parts of the head [15-16, 37, 39, 43], scapular, lateral brachial, and latissimus dorsi flaps for deep soft tissue defects [15, 16, 39, 43], and iliac crest, fibular, and scapular flaps for maxillary and mandibular defects. Furthermore, repair of large maxillo-facial defects can be augmented with an implantable prosthesis for optimal outcome [39].

However, restoration of bony defects is not always an option for pediatric patients since the autograft can halt the growth of the recipient bone [42]. Moreover, Yano, et al. suggest that osteotomies of the maxilla and mandible cause little disruption to overall facial bone development [41]. Hence, bony reconstruction is ideally performed on adults. For instance, Korfage, et al. presented a case of a 12-year-old who was successfully cured of parameningeal RMS, after extensive debulking combined with postoperative chemotherapy [44]. The treatment had resulted in mid-facial hypoplasia from abnormal bone growth [44]. The defect improved significantly with rostral advancement of the mid-face by 1.5 cm over a month by an external distraction frame (ID: lens.org/114-884-051-888-872) in combination with prosthodontics, reconstruction of the palate with temporalis muscle, and rehabilitation [44].

A novel reconstruction technique: use of a perforator-based flap

In the perforator-based flap technique, terminal cutaneous branches, which are thinly encircled with only small amounts of fat and muscle, are salvaged from the donor site [43]. This thin flap is transplanted to cover flat defects, which are very common in the oral mucosa or facial skin, and produces an optimal esthetic outcome [43].

The importance of skull base reconstruction and its current advances

Skull base defects are common sequelae of resection for parameningeal RMS [36]. Prompt reconstruction is required to prevent associated complications and mortality [45]. Such reconstruction is relevant to almost all patients who have undergone excision of high-grade parameningeal HNRMS [35, 46]. Regional or free flaps are used to carry out this procedure safely [35]. For instance, the temporalis muscle can be used in skull base surgery for patients with parameningeal HNRMS and gives a satisfactory result [45]. Moreover, Gil, et al. conducted a study across multiple healthcare institutions and discovered that most complications in pediatric skull base reconstruction were associated only with local wound healing [40].

Hayashi, et al. introduced an interesting technique of using the peri-fascial alveolar tissue (which is highly flexible), claimed from the inguinal or femoral region, for complication-free repairs of the skull base [47]. Only one patient out of 14 enrolled, i.e., 4.8%, had a further CSF leak [40, 47].

The novel concept of palliative surgery and its potential importance in HNRMS patient care

Very few papers discuss the importance of palliative surgery. Although locally advanced and metastatic diseases may not have a surgical "cure," surgical "palliation" is certainly valuable since patients with local recurrence or distant metastasis often survive up to 72 months [45]. For example, Weizman, et al. performed palliative surgery on a child with a relapsed local disease and another with a metastatic disease, who both benefited from functional restoration and improved quality of life [39]. Cantu, et al. added a study of multiple cases in which surgical debulking of a skull base tumor provided significant symptomatic relief and a favorable quality of life regardless of grade [45].

Although the COG recommends that high-grade tumors should be dealt with conservatively, it is important to discuss the option of palliative surgery with the patient and parents to prolong the disease-free time [27, 45, 48].

Discussion

**Figure 2:**
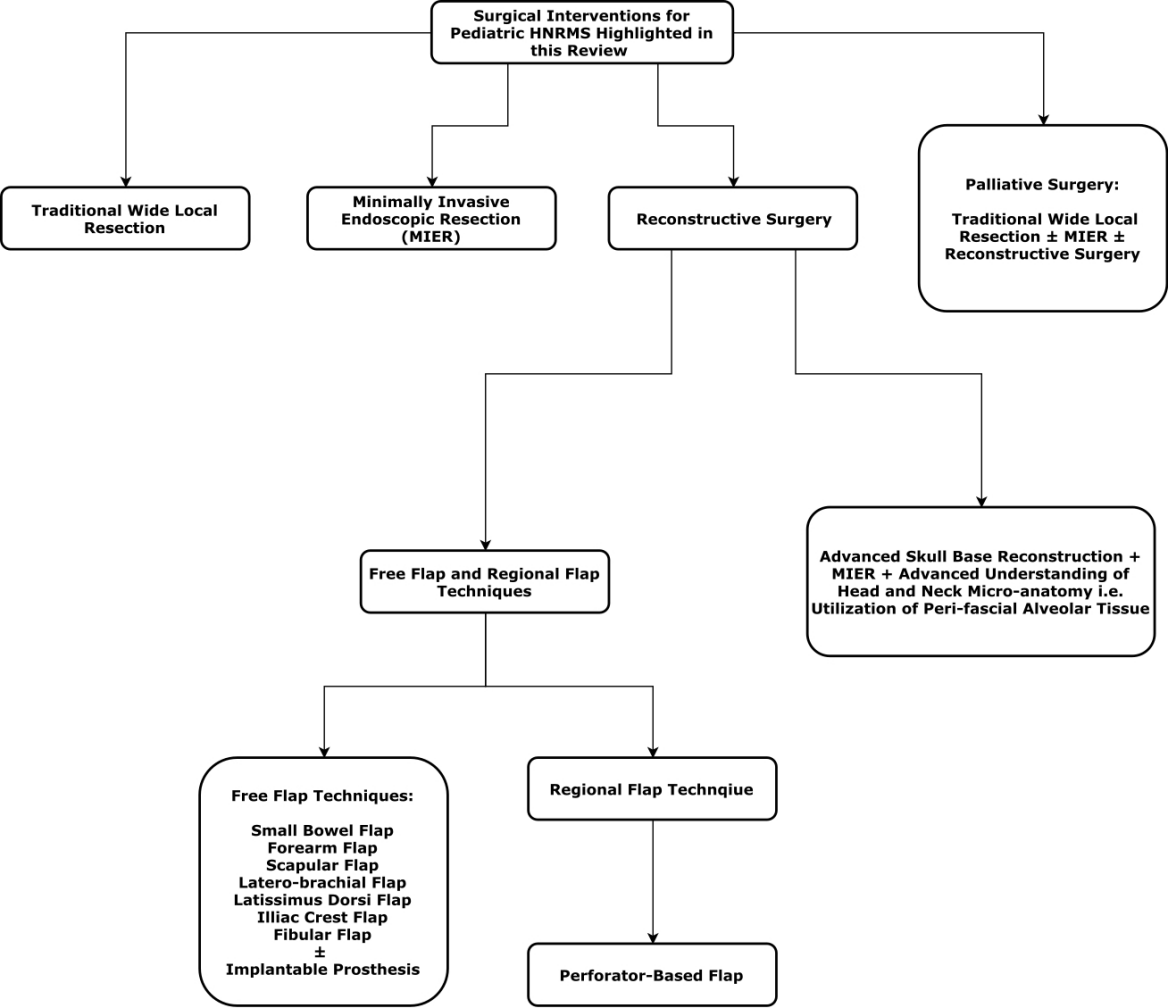
A summary of surgical interventions for pediatric HNRMS HNRMS; rhabdomyosarcoma of the head and neck, MIER; minimally invasive endoscopic resection

Traditionally, resection with a clear margin followed by immediate reconstruction has been the key to surgical management of RMS [15]. However, HNRMS poses a great technical challenge to favorable resection [14, 15, 19, 20, 28, 33]. Although it is very challenging, recent advances in surgical techniques and the better understanding of microanatomy now enable surgeons to conduct operations beyond what was previously considered possible, i.e., surgical exploration of the parameningeal area (Figure 2).

For the past three decades, despite a significant improvement in five-year survival rate from 25% to 87%, which was achieved by implementing IRSG/COG protocols in RMS management, locally advanced, relapsed, and metastatic diseases are still considered difficult to treat in the field of head and neck oncology [8, 22].

Researchers have struggled to discover and translate novel biological targets into targeted therapies for RMS [1, 5, 12, 30, 32]. Moreover, VAC has been the constant chemotherapeutic regime since the 1960s [1, 2, 12]. Interestingly, most clinical trials for RMS have focused on understanding the genetics and biological pathogenesis of RMS and testing novel treatments, but very few studies have focused on improving surgical options for HNRMS [5, 8, 30, 31]. This uneven distribution of interests could have been aggravated by “the real breakthrough” [8] during the 1960s when the first chemotherapeutics improved the survival rate from 5%-9% (prior to 1969) to 25% in the 1970s; arguably the first and most significant achievement in the history of RMS treatment [2, 3, 5, 8, 14, 16-17, 30].

Currently, although “operability” is the single most important assessment tool for survival in RMS, there is a lack of and need for a standardized surgical protocol for HNRMS, which should cover the use of endoscopic technology, reconstructive microsurgery, and palliative surgery. Development of this protocol would further clarify surgery's critical role in improving the patient’s quality of life [36].

There is still a need for larger cohort studies comparing the efficacies of endoscopic and non-endoscopic approaches in achieving a disease-negative margin in HNRMS, developing and/or deploying novel surgical techniques, assessing prosthetic implants, rehabilitation devices for HNRMS, and further exploring the novel reconstruction techniques, e.g., the perforator-based flap technique described by Hölzle, et al. [39, 43].

A standardized surgical protocol for HNRMS and the potential study topics described above would also be applicable to managing non-RMS tumors of the head and neck such as Ewing's sarcoma, neuroblastoma, and squamous cell carcinoma [32, 36, 38, 46, 49]. Moreover, advances in head and neck reconstruction technology would be useful in managing traumatic facial injury such as loss of tissue and repairing burns wounds [43].

## Conclusions

This study comprehensively reviews currently available surgical techniques and options for advanced pediatric parameningeal HNRMS. Although surgery is often the most important element in managing this disease, it is also the most challenging. We highlight the need for a surgical protocol, which addresses all currently available surgical options, and large cohort studies to explore novel surgical intervention for this disease. Such an effort would aid in providing the most individualized interventions to patients. It would also help dispense sufficient information about surgical risks and how they could be minimized and prevented, with the use of modern technologies and available clinical data, to parents and older patients. Most importantly, the procedure must be performed in the best interest of the patient and parents, not for indulging in one’s egoism. The goal is to provide children and adolescents with improved quality of life and minimized treatment-associated adverse events, which can seriously affect one’s remaining decades of life.
